# TRPV1-Mediated Microglial Autophagy Attenuates Alzheimer’s Disease-Associated Pathology and Cognitive Decline

**DOI:** 10.3389/fphar.2021.763866

**Published:** 2022-01-18

**Authors:** Chenfei Wang, Wei Huang, Jia Lu, Hongzhuan Chen, Zhihua Yu

**Affiliations:** ^1^ Department of Pharmacology and Chemical Biology, Shanghai Jiao Tong University School of Medicine, Shanghai, China; ^2^ Cardiology Department, The First Affiliated Hospital of Chongqing Medical University, Chongqing, China; ^3^ Shanghai University of Traditional Chinese Medicine, Shanghai, China

**Keywords:** TRPV1, microglia, autophagy, capsaicin, metabolism

## Abstract

Autophagy is a major regulator of the ageing process of the central nervous system and neurodegeneration. Autophagy dysfunction has been implicated in the pathogenesis of Alzheimer’s disease (AD). TRPV1 was reported to regulate autophagy to protect against foam cell formation and reduce the release of inflammatory factors in atherosclerosis. In this study, pharmacological activation of TRPV1 with the TRPV1 agonist capsaicin induced autophagy in a TRPV1-dependent manner in both primary microglia and BV2 cells. TRPV1-mediated autophagy regulated glycolysis and oxidative phosphorylation by controlling the expression of genes required for aerobic glycolysis and mitochondrial respiration in primary microglia. TRPV1 agonist capsaicin decreased amyloid and phosphorylated tau pathology and reversed memory deficits by promoting microglia activation, metabolism, and autophagy in 3xTg mice. These results indicate that TRPV1 was a potential therapeutic target for AD, which suggests that capsaicin should be further assessed as a possible treatment for AD.

## Introduction

Autophagy is a major regulator of the ageing process of the central nervous system and neurodegeneration, during which toxic molecules and organelles aggregate in intracellular autophagosomes and are then degraded in lysosomes (Menzies et al., 2015). Autophagy dysfunction has been implicated in the pathogenesis of neurodegenerative disorders, including Alzheimer’s disease (AD) (Metaxakis et al., 2018). Upregulation of mechanistic target of rapamycin (mTOR) kinase, an important regulator of the autophagy pathway, is associated with reduced autophagy in AD patients (Caccamo et al., 2010). Deletion of the beclin 1 (BECN1) gene, which codes for a key autophagy-related protein (ATGs) in autophagosome formation, is reported to increase both the intracellular and extracellular deposition of amyloid β (Aβ) plaques in animal models of AD (Swaminathan et al., 2016). Therefore, induction of autophagy might be one of the promising therapeutic strategies for the prevention and treatment of AD. Mounting studies have evaluated the role of autophagy on neuron in neurodegeneration, emerging evidence shows that autophagy may also be involved in microglia immune function (Dello Russo et al., 2013).

Microglia, akin to tissue resident macrophages, are brain immune cells that are important for central nervous system homeostasis including modulating learning and memory, monitoring neuron activity, and acting as local phagocytes (Hanisch and Kettenmann, 2007). Microglia can respond to brain damage and engulfing brain-derived cargo including axonal fragments and protein deposits such as Aβ plaques. In the peripheral immune system, autophagy was shown to involve innate and adaptive responses (Hanisch and Kettenmann, 2007), antigen presentation process, and phagocytosis (Münz, 2016). Both immune tolerance and chronic inflammation during aging have shown to correlate with autophagy flux impairment in macrophages (Stranks et al., 2015). While few studies have evaluated the role of autophagy on microglia immune function including phagocytosis and inflammation, as well as the possible impact of autophagy dysregulation of these brain-resident macrophages during the pathological condition such as AD.

Transient receptor potential vanilloid type 1 (TRPV1) was expressed in microglia and activation of TRPV1 triggered an increase in intramitochondrial Ca^2+^ concentration and following of mitochondria depolarization which resulted in enhancement of chemotactic activity in microglia (Ji et al., 2002; Costa et al., 2004). Capsaicin-induced activation of TRPV1 causes the activation of peroxisome proliferator-activated receptor δ, which significantly increases the expression of ATGs *in vitro* and reduces the release of inflammatory factors in a wildtype (WT), but not TRPV1^−/−^, mouse model of nonalcoholic fatty liver disease (Li et al., 2013). Moreover, TRPV1-mediated autophagy rescued mouse cardiac function in a mouse model of streptozotocin-induced diabetes mellitus (Tong et al., 2018).

In this study, we hypothesized that TRPV1-mediated microglial autophagy would reduce amyloid and phosphorylated tau pathology and attenuate cognitive decline in a mouse model. TRPV1-mediated autophagy regulated glycolysis and oxidative phosphorylation in microglia by controlling the expression of genes required for aerobic glycolysis and mitochondrial respiration. Cellular uptake of Aβ_1-42_ was facilitated by TRPV1 activation in microglia. To explain the potential beneficial effect of a TRPV1 agonist in AD, the use of 3xTg mice models demonstrated that TRPV1 was a potential therapeutic target for AD and TRPV1-mediated microglial autophagy.

## Methods

### Mice

3xTg transgenic mice (no. 003378) were purchased from The Jackson Laboratory (Bar Harbor, ME, United States). TRPV1^−/−^ mice with a C57BL/6J background were kind gifts from Wei Huang (Department of Cardiology, The First Affiliated Hospital of Chongqing Medical University, Chongqing, China). The mice were housed at room temperature (22 ± 1°C) under a 12-h light/dark cycle. The protocols of all animal experiments were approved by the Animal Experimentation Ethics Committee of Shanghai Jiao Tong University School of Medicine (Shanghai, China).

### Capsaicin Treatment

Capsaicin was purchased from Target Molecule (TargetMol, Corp. Shanghai, China). Seven-month-old male and female littermate wildtype (WT) and 3xTg mice were injected with capsaicin (1 mg/kg, intraperitoneally; a single injection/day for 1 month) (Veldhuis et al., 2003; Nam et al., 2015). Behavioral tests were carried out 24 h later following the final injection of capsaicin. Afterward, the mice were euthanized and brain tissues were collected for Western blots and histological analyses.

### BV2 Cell Culture

Cells from the murine microglial BV2 cell line were purchased from ATCC. BV2 cells were cultured in Dulbecco’s modified Eagle’s medium (DMEM, Hyclone SH30022.01B, Beijing, China) with 10% fetal bovine serum, 2 mM L-glutamine (Beyotime C0212, Shanghai, China), 100 U/mL penicillin, and 0.1 mg/ml streptomycin (Beyotime C0222, Shanghai, China) under standard culture conditions (37°C with 5% CO_2_ and 95% relative humidity).

### Primary Microglia Culture

Primary mixed glial cells were cultured from brain tissue from newborn WT or TRPV1^−/−^ mice (P0-P2). Briefly, after removing the meninges, cerebral cortices were dissected into small pieces and digested by 0.125% trypsin (Beyotime C0203-100ml, Shanghai, China) for 15 min at 37°C with mechanical disruption. Cells were cultured in DMEM with 10% fetal bovine serum, 2 mM L-glutamine, 100 U/mL penicillin, and 0.1 mg/ml streptomycin at 37°C with 5% CO_2_ for 10–14 days. Then the mixed cells with media were shaken at 200 rpm for 4 h at 37°C to suspend loosely attached microglia. The media was centrifuged, and the microglia were pelleted.

### Transmission Electron Microscopy

Cells were fixed in 2.5% glutaraldehyde in phosphate-buffered saline (PBS) for 4 h at 4°C, then scraped gently and collected by centrifugation. The samples were rinsed 2 times with PBS for 15 min and post-fixed in 1% osmium tetroxide for 2 h at 4°C, and then rinsed again. After that, the cells were dehydrated by an increasing gradual set of ethanol and then embedding in Epon 812 for 48 h at 37°C. Ultra-thin sections were cut by LEICA Ultracut R and stained with uranyl acetate and lead citrate, then observed under transmission electron microscope (TEM) Hitachi H-7650 (Hitachi, Tokyo, Japan). The length and area of mitochondria were determined using FIJI software (NIH, United States).

### Cell Metabolism Analysis

Cell metabolism was measured by a Seahorse XFe cell analyzer (Agilent Technologies, Inc., Santa Clara, CA, United States) as previously described (Lu et al., 2021). Shortly, BV2 cells were plated in the XF96 cell culture microplates at 1 × 10^5^ cells/well, and after adherence overnight. After the drug administration, cells were washed and then incubated in Seahorse XF Base medium (102353–100; Seahorse Bioscience) supplemented with 25 mM glucose, 200 mM glutamine, and 1 mM pyruvate. The oxygen consumption rate (OCR) was performed using 5 μM oligomycin, 10 μM carbonyl cyanide p- (trifluoromethoxy) phenyl-hydrazone (FCCP), and 10 μM rotenone and antimycin A to measure mitochondrial respiration. The extracellular acidification rate (ECAR) was detected by an XF glycolysis stress test kit, including 10 mM glucose, 0.5 μM oligomycin, and 50 mM 2-deoxy-glucose (2-DG). Results were analyzed and exported using Wave 2.6.0 version (Agilent Technologies, Inc.).

### Measurement of Autophagy Flux

The autophagy levels were measured as previously reported (Lu et al., 2021). In brief, the BV2 cells and primary microglia were seeded at a density of 1 × 10^5^ cells/well and cultured overnight, after which cells were transfected with mRFP-GFP-LC3 plasmids (Addgene, Inc., Watertown, MA, United States) for 48 h according to the manufacturer’s protocol (Guangzhou RiboBio Co., Ltd, Guangzhou, China). Media was aspirated and then cells were incubated with 10 μM capsaicin or 2 μM rapamycin for 24 h. Cells were fixed with 4% paraformaldehyde and the digital images of LC3-positive puncta were captured by TCS SP8 confocal laser scanning microscope (Leica Microsystems GmbH). The numbers of LC3-positive puncta per cell in each experimental group were counted with ImageJ software.

### Cellular Uptake Study

BV2 cells and primary microglia were cultured in a 96-well plate at a density of 1 × 10^4^ cells/well overnight, and then treatment with 10 μM capsaicin in serum-free DMEM for 24 h. For quantification of cellular uptake, cells were incubated with fluorescein isothiocyanate (FITC)-conjugated Aβ_1–42_ (FITC-Aβ_1–42_, GenicBio Biotechnology Co., Ltd., Shanghai, China) on a concentration of 2 μg/ml for 4 h; next, the cells were fixed with 4% paraformaldehyde and the nuclear stained with 4′,6-diamidino-2-phenylindole, dihydrochloride (DAPI). The microglial phagocytic activity of FITC-Aβ_1–42_ was quantitatively detected using a Cellomics KineticScan reader (Thermo Fisher Scientific).

### Western Blotting

Cultured cells and the mice hemibrain tissue were lysed in ice-cold RIPA lysis buffer (Beyotime, P0013B) containing 1 mM phenylmethylsufonyl fluoride. The homogenates were centrifuged for 10 min at 4°C. The supernatants were subsequently boiled at 100°C for 10 min after adding 5× sodium dodecyl sulfate loading buffer. There were 50 μg protein samples separated by sodium dodecyl sulfate-polyacrylamide gel electrophoresis, and then proteins were transferred to a polyvinylidene difluoride membrane. The membrane was blocked in tris-buffered saline containing 5% skim milk at room temperature for 1 h. After being incubated with primary antibodies (Table 1) at 4°C overnight, the membranes were then incubated with the peroxidase-conjugated anti-rabbit (A0208, Beyotime Institute of Biotechnology) or anti-mouse (A0216, Beyotime Institute of Biotechnology) IgG antibody 1 h at room temperature. The immunoreactive proteins were visualized by the enhanced ECL chemiluminescent substrate kit (Yeasen, 36222ES76), and measured by Image Studio Lite Ver 5.2 software (LI-COR Biosciences, Lincoln, NE, United States).

**TABLE 1:** Primary antibodies used in this study

Primary antibodies	Source	Catalog no.	Western blot	Immuno-fluorescence
Rabbit monodonal anti-Phospho-mTOR (Ser2448)	Cell signaling technology	5536T	1:1000	—
Rabbit monodonal anti- mTOR	Cell Signaling Technology	2983T	1:1000	—
Rabbit monodonal anti-Phospho-AMPKα (Thr172)	Cell Signaling Technology	2535T	1:1000	—
Rabbit monodonal anti-Phospho-AKT (Thr308)	Cell Signaling Technology	2965P	1:1000	—
Rabbit monodonal anti- AKT	Cell Signaling Technology	4691P	1:1000	—
Rabbit polydonal anti-LC3B	Cell Signaling Technology	2775	1:1000	—
Mouse monodonal anti-LC3B(E5Q2K)	Cell Signaling Technology	83506S	-	1:400
Rabbit polydonal anti-SQSTM1	Cell Signaling Technology	5114S	1:1000	—
Mouse monodonal anti-HIF-1α	Beyotime Institute of Biotechnology	AH339-1	1:500	—
Rabbit monodonal anti-PSD95 (D74D3)	Cell Signaling Technology	3409	1:1000	—
Mouse monodonal anti-GFAP (GA5)	Cell Signaling Technology	3670T	1:1000	—
Rabbit polydonal anti-Iba1	Wako Pure Chemical Industries	019–19741	1:1000	1:200
Rabbit monodonal anti-TRPV1 (VR1)	Alomone labs	ACC-030	1:200	—
Rabbit monodonal anti-Beclin-1 (D40C5)	Cell Signaling Technology	3495T	1:1000	—
Rabbit monodonal anti-Atg16L1 (D6D5)	Cell Signaling Technology	4445T	1:1000	—
Rabbit monodonal anti-Atg7 (D12B11)	Cell Signaling Technology	4445T	1:1000	—
Mouse monodonal anti-Phospho-Tau (Ser396) (PHF13)	Cell Signaling Technology	9632S	1:1000	1:500
Mouse monodonal anti-Tau (clone 46.1)	Millipore	2480581	1:1000	—
Mouse monodonal anti-β-actin	Cell Signaling Technology	3700S	1:1000	—
Rabbit monodonal anti-NeuN	Beyotime Institute of Biotechnology	AF1072	1:1000	1:100
Mouse monodonal anti-human Aβ (clone 6E10)	Bio Legend	800701	1:1000	1:500

### Immunofluorescence

Mouse brain sections were prepared as described previously (Yi et al., 2016a; Yi et al., 2016b). Briefly, mice were perfused with 0.9% NaCl solution and 4% paraformaldehyde successively after anaesthetization. Then brains were removed for immersion in 4% paraformaldehyde and dehydration by sucrose, and were embedded in Tissue-Tek O.C.T. compound (Sakura Finetek United States 4583), then sectioned 20 μm-thick coronally using a freezing microtome (Leica CM 1950, Wetzlar, Germany). Sections were blocked in PBS with 10% normal goat serum and 0.3% Triton-X for 30 min. Sections were incubated with primary antibodies (Table 1) at 4°C overnight, then incubated with secondary antibodies at room temperature for 2 h. Secondary antibodies were Alexa Fluor 488-conjugated anti-mouse IgG (1:500) and Alexa Fluor 555-conjugated anti-rabbit IgG (1:500). Finally, brain sections were stained nucleus by DAPI (1:1000) for 15 min and glass covers. Immunofluorescent images were visualized and captured using Leica TCS SP8 confocal laser scanning system (Leica Microsystems, Wetzlar, Germany). For all analyses, at least three images were taken in each brain region, and slides used ×40 oil objective, at 1024 × 1024 resolution, with *z*-step size of 0.99 μm at 13.8 μm. For the quantification of colocalization of LC3B and Iba-1, LC3B and NeuN, or p-tau (Ser396) and NeuN, FIJI 2.0 plugin coloc2 were utilized. In brief, images were imported to FIJI, and data channels were separated. Then FIJI plugin coloc2 was switched on, and Manders’ colocalization index was calculated and recorded. For the quantification of 4G8^+^ β-amyloid, data of fluorescent area was measured and recorded.

### Two-Dimensional Morphological Analysis of Microglia

Two-dimensional morphological analysis of microglia was performed using ImageJ as described previously (Lu et al., 2021). Briefly, 20 microglial cells per group that randomly selected from both cerebral cortex and hippocampus were segmented into single-cell using a custom-written ImageJ plugin. Single-cell images were automatically converted to 8-bit and transformed into binary images by application of automatically calculated intensity threshold. The parameters “Mean branch length” and “end-point voxels” were quantified using FIJI plugins “Skeletonize” and “Analyze skeleton 2D/3D” to skeletonize and analyze the binary single-cell images obtained in the previous step (Young and Morrison, 2018).

### Novel Object Recognition Assessment

Novel object recognition assessment was conducted on two successive days. On Day 1, mice were placed in a corner of an empty mouse open field box and allowed to explore for 8 min. On Day 2, mice were placed in the open field box with the two same objects and allowed to explore for 8 min 4 h later, mice were exposed again to a novel and a familiar object and allowed to explore for 8 min. Exploring activities, including facing, touching, and/or sniffing the object, were recorded and analyzed in SuperMaze, and the percent of the time mice spent exploring the novel object was calculated.

### Y Maze Spontaneous Alteration Test

Y maze spontaneous alteration test included 2 sections. In the first section (training trial), mice were placed into one arm (start arm) and allowed to explore in two arms for 5 min. After 1 h interval, the third arm was opened as a novel arm. In the second section (test trial), mice were placed in the Y maze and allowed to explore all three arms for 5 min. Activities of mice in the Y maze were recorded in SuperMaze, and spontaneous alteration (percent) was calculated, which was defined as the number of times to novel arm divided by total arm alterations minus 2 × 100.

### Morris Water Maze Behavioral Assessment

Morris water maze behavioral assessment was performed as previously described (Wei et al., 2019). Briefly, the tests were performed in a 120 cm diameter, 45 cm deep circular pool with opacified water kept at 20 ± 1°C. An escape platform whose diameter is 4.5 cm was placed 1 cm below the surface of the water. Recording and analyzing system (Shanghai Jialiang Software Technology Co, Ltd.) was also applied. Training consisted of daily sessions (4 trials per session) for 6 consecutive days. Mice were put into the pool from 4 pseudorandomly varied cardinal points. Each mouse was required to find the hidden platform within 60 s and offered 60 min intertrial interval. Mice would be placed on the platform for an extra 10 s if they did not find the platform in 60 s. After 6-days’ training, the platform was no longer presented and the mice took a probe trial, in which each mouse was put into the pool from two pseudorandomly varied cardinal points and performed for 60 s. Behavioral characteristic (swim distance, swim speed, latency, time spent in each quadrant, and number of times of platform crosses) was recorded and analyzed.

### RNA Sequencing Analysis

RNA isolation and purification, cDNA library construction, and sequencing were performed as previously described (Yu et al., 2017). Briefly, total RNA was isolated from the hemibrains of mice with TRIzol reagent (Invitrogen Life Technologies) and treated with DNase to remove DNA. RNA quantity and quality were determined with a NanoDrop spectrophotometer (Thermo Scientific). mRNA was isolated from total RNA with poly-T oligonucleotide-attached magnetic beads. Ion interruption was used to interrupt RNA into fragments of about 300 bp. The first cDNA strand was synthesized by using 6-base random primers, after that, the second-strand cDNA was synthesized by adding buffer solution, dNTPs, RNase H, and DNA polymerase I. The RNA library was sequenced on the Illumina Hiseq platform by Shanghai Personal Biotechnology Cp. Ltd. Hierarchical clustering of differentially expressed genes was performed using the R language Pheatmap (1.0.8) software package. Gene Ontology (GO) enrichment analysis was performed to determine the main biological functions performed by the differential genes. According to the GO enrichment result, the enrichment degree is measured by Rich factor, FDR value, and the number of genes enriched in this GO Term. The top 20 GO Term which entries the smallest FDR value were displayed. Meanwhile, Kyoto Encyclopedia of Genes and Genomes (KEGG) enrichment analysis was performed via https://www.genome.jp/kegg/ to determine the signaling pathways that the differential genes mainly participate in.

### Weighted Gene Co-Expression Network Analyses

Weighted gene co-expression network analyses (WGCNA) were performed to analyze 3xTg mice brain co-expression networks that were changed after capsaicin treatment (Zhang and Horvath, 2005). Co-expression networks were built by WGCNA package in R (Langfelder and Horvath, 2008). Considering the equilibration between power and scale independence, the power of 12 was chosen to build scale-free topology for all datasets. The mean of the trajectory of the module eigengenes (MEs) of given modules was calculated by first taking the row sum of the adjacency matrix as the intra modular connectivity score per gene, and then the average of the intra modular connective score in the given modules was calculated. Genes in given modules were enriched into pathways of both GO and KEGG using Metascape (https://metascape.org) (Zhou et al., 2019), and selected networks were visualized by Cytoscape v3.8.0 (Shannon et al., 2003).

### Metabolite Profiling

Liquid chromatography–mass spectrometry analyses were conducted on a Q Exactive™ Hybrid Quadrupole-Orbitrap™ Mass Spectrometer equipped with an Ion Max™ source and a Heated Electrospray Ionization (HESI-II) Probe, which was coupled to a Dionex UltiMate™ 3000 ultra-high-performance liquid chromatography system (Thermo Fisher Scientific). External mass calibration was performed, using a standard calibration mixture, every 7 days. For metabolite profiling, microglia were stimulated with Earle’s balanced salts solution (EBSS) for 24 h. Cells were collected and centrifuged. Then, the cell pellets were washed with ice-cold PBS. Polar metabolites were extracted using 1 ml of ice-cold 80% methanol with 10 ng/ml of valine-d_8_ as an internal standard. After vortexing for 10 min and centrifugation for 10 min at 4°C and 10,000 × *g*, the samples were dried in a table-top vacuum centrifuge. The dried samples were stored at 80°C. Prior to analysis, the samples were resuspended in 100 µL of water and 1 µL of each sample was injected onto a SeQuant^®^ ZIC^®^-pHILIC 2.1 × 150 mm (5 µm particle size) column (EMD Millipore Corporation). Buffer A consisted of 20 mM ammonium carbonate and 0.1% ammonium hydroxide, whereas buffer B was acetonitrile. The chromatographic gradient was run at a flow rate of 0.15 ml/min as follows: 0–20 min: linear gradient from 80 to 20% B; 20–20.5 min: linear gradient from 20 to 80% B; and 20.5–28 min: hold at 80% B. The mass spectrometer was operated in full-scan, polarity switching mode with the spray voltage set at 3.0 kV, the heated capillary held at 275°C, and the HESI-II probe held at 350°C. The sheath gas flow was set to 40 U, the auxiliary gas flow was set to 15 U, and the sweep gas flow was set to 1 U. MS data acquisition was performed in a range of 70–1000 m/z, with the resolution set at 70,000, the automatic gain control target at 10^6^, and the maximum injection time at 80 ms. Relative quantitation of polar metabolites was performed with Xcalibur™ QuanBrowser 2.2 software (Thermo Fisher Scientific) with a mass tolerance of 5 ppm and reference to an in-house library of chemical standards. The relative abundance of metabolites was calculated by normalizing the profiling results to the cell number.

### Real-Time PCR

Primary microglia were frozen in TRIzol solution (Invitrogen Corporation). Total RNA was isolated and transcribed to cDNA using a RevertAid^TM^ First-Strand cDNA Synthesis Kit (Fermentas, Glen Burnie, MD, United States), according to the manufacturer’s protocol. Quantitative real-time PCR (qPCR) was performed on an ABI 7500 sequence detector (Applied Biosystems) using SYBR Green I. The primer sequences were as follows: for *Slc2a1,* 5′-GAG​ACC​AAA​GCG​TGG​TGA​GT-3’ (sense) and 5′-GCA​GTT​CGG​CTA​TAA​CAC​TGG-3’ (antisense); for *Slc2a3*, 5′-ATC​GTG​GCA​TAG​ATC​GGT​TC-3’ (sense) and 5′-TCT​CAG​CAG​CTC​TCT​GGG​AT-3’ (antisense); for *Irf4*, 5′-CAA​AGC​ACA​GAG​TCA​CCT​GG-3’ (sense) and 5′-TGC​AAG​CTC​TTT​GAC​ACA​CA-3’ (antisense); for *Hif1a*, 5′-AAA​CTT​CAG​ACT​CTT​TGC​TTC​G-3’ (sense) and 5′-CGG​CGA​GAA​CGA​GAA​GAA-3’ (antisense); for *Hk2*, 5′-TGA​TCG​CCT​GCT​TAT​TCA​CGG-3’ (sense) and 5′-AAC​CGC​CTA​GAA​ATC​TCC​AGA-3’ (antisense); for *Gpi*, 5′-TCA​AGC​TGC​GCG​AAC​TTT​TTG-3’ (sense) and 5′-GGT​TCT​TGG​AGT​AGT​CCA​CCA​G-3’ (antisense); for *Pkfl*, 5′-GGA​GGC​GAG​AAC​ATC​AAG​CC-3’ (sense) and 5′-CGG​CCT​TCC​CTC​GTA​GTG​A-3’ (antisense); for *Aldoa*, 5′-CGT​GTG​AAT​CCC​TGC​ATT​GG-3’ (sense) and 5′-CAG​CCC​CTG​GGT​AGT​TGT​C-3’ (antisense); for *Pgk1*, 5′-ATG​TCG​CTT​TCC​AAC​AAG​CTG-3’ (sense) and 5′-GCT​CCA​TTG​TCC​AAG​CAG​AAT-3’ (antisense); for *Pgam1*, 5′-TCT​GTG​CAG​AAG​AGA​GCA​ATC​C-3’ (sense) and 5′-CTG​TCA​GAC​CGC​CAT​AGT​GT-3’ (antisense); for *Eno1*, 5′-TGC​GTC​CAC​TGG​CAT​CTA​C-3’ (sense) and 5′-CAG​AGC​AGG​CGC​AAT​AGT​TTT​A-3’ (antisense); for *Pkm2*, 5′-GCC​GCC​TGG​ACA​TTG​ACT​C-3’ (sense) and 5′-CCA​TGA​GAG​AAA​TTC​AGC​CGA​G-3’ (antisense); for *Gapdh*, 5′-TGT​GTC​CGT​CGT​GGA​TCT​GA-3’ (sense) and 5′-AGG​GGC​CAT​CCA​CAG​TCT​TC-3’ (antisense). Analysis by qPCR included the following steps: a hold step at 50°C for 2 min to activate uracil-DNA glycosylase with a second hold step at 95°C for 10 min, followed by 40 cycles at 95°C for 15 s followed by 60°C for 1 min. Subsequently, melt analysis was performed by increasing the temperature from 65°C to 95°C. Target gene expression was normalized to GAPDH using the 2^-∆∆CT^ method.

### Statistical Analysis

All data are presented as mean ± standard error of the mean. Data were tested for Gaussian distribution with the Kolmogorov-Smirnov normality test and then analyzed by one-way analysis of variance (ANOVA) and Tukey’s post hoc test. Data were analyzed with an unpaired, two-tailed Student’s t test when comparing between two groups; if the variable failed the normality test, the non-parametric Mann-Whitney U test was applied. A *p* value < 0.05 was considered statistically significant. All those statistical analyses were performed using Prism 7 (Graphpad Software, Inc., LA Jolla, CA, United States).

## Result

### TRPV1 Activation Induced Autophagy and Energetic Metabolism in Microglia

**FIGURE 1:**
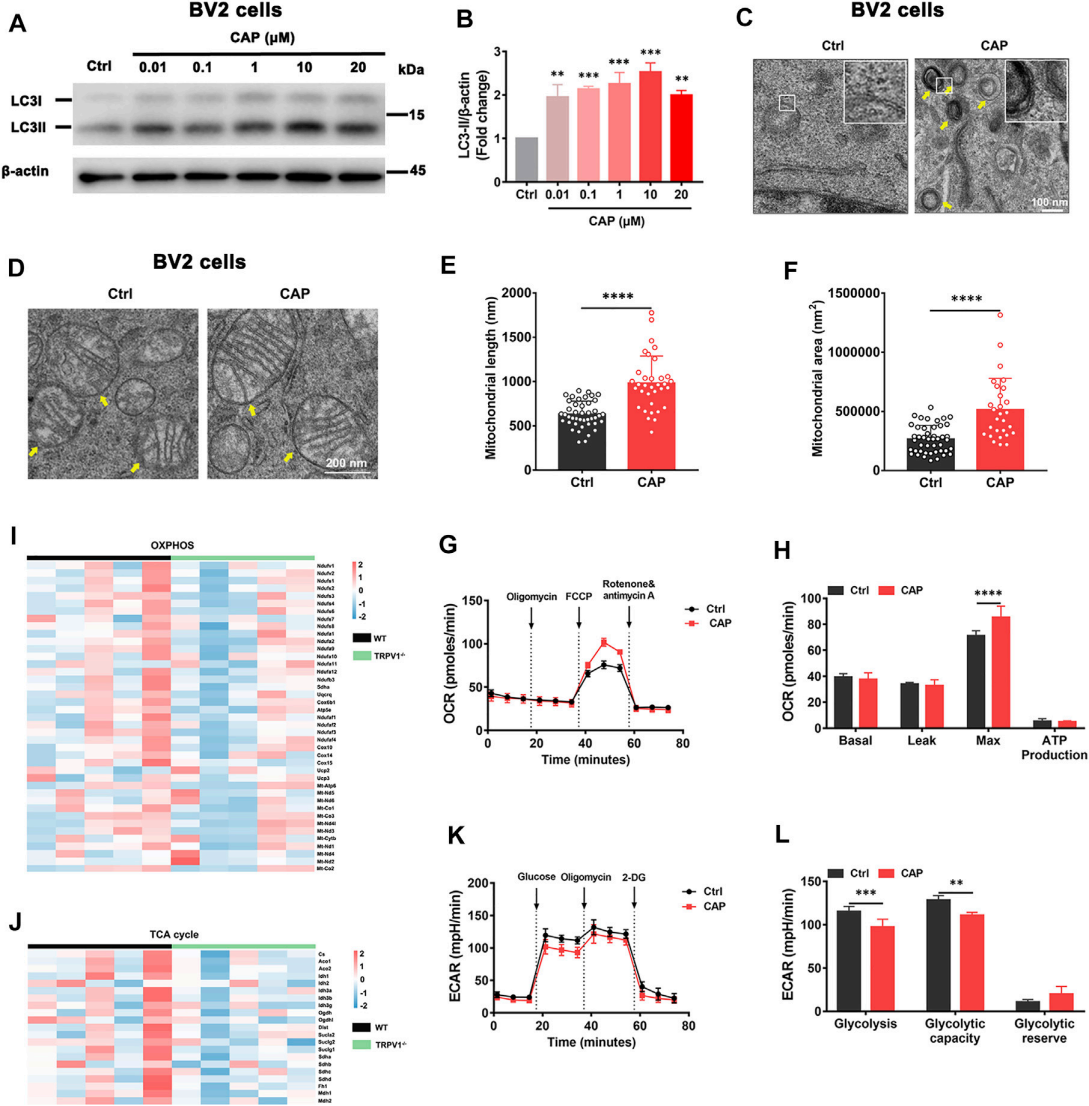
TRPV1 agonist induced autophagy and energetic metabolism in microglia. **(A, B)** LC3-II/I protein expression in BV2 cells with 0.01, 0.1, 1, 10, or 20 μM capsaicin treatment for 24 h. Relative protein abundance was normalized to β-actin (*n* = 3, three independent experiments). **(C, D)** The formation of autophagosomes and the morphology of mitochondria were observed by transmission electron microscopy in BV2 cells with 10 μM capsaicin treatment. **(E, F)** The length and area of mitochondria in BV2 cells after being treated with 10 μM capsaicin (*n* = 3, three independent experiments). **(G, H)** Mitochondrial respiratory function reflected by OCR was detected in BV2 cells with 10 μM capsaicin treatment (*n* = 6, three independent experiments). **(I, J)** Heatmaps represent relative expression of the OXPHOS genes and the TCA cycle genes in brains of WT (*n* = 5) and TRPV1^−/−^ (*n* = 5) mice. (**K, L**) Glycolytic capacity calculated by ECAR in BV2 cells with 10 μM capsaicin treatment (*n* = 6, three independent experiments). ^**^ *p* < 0.01, ^***^ *p* < 0.001, ^****^ *p* < 0.0001, one-way ANOVA with the Tukey’s post-hoc test. Bars represent mean ± SEM.

To determine the impact of TRPV1 channels on microglial autophagy, the autophagy marker and the structure of microglia were examined following treatment with capsaicin, a TRPV1 agonist, by Western blot and transmission electron microscopy. Upregulated expression of the microtubule-associated protein 1 light chain 3 beta (MAP1LC3B, LC3B-II) and LC3B-I proteins was observed in BV2 cells treated with 0.01, 0.1, 1, 10, or 20 μM capsaicin for 24 h, in a dose-dependent manner (Figures 1A,B). Strikingly, abundant multilamellar structures suggestive of autophagosomes were found in BV2 cells treated with 10 μM capsaicin, but not in the control cells (Figure 1C). Given that autophagy plays an important role in metabolism and cell survival of microglia (Lee et al., 2019), we then examined mitochondrial morphology and energy metabolism state of microglia. Mitochondrial length and mitochondrial area were increased in the capsaicin group compared to the control group (Figures 1D–F). The OCR represented mitochondrial function and assessed under sequential treatment of oligomycin, FCCP, rotenone, and antimycin A. Capsaicin treatment increased the maximal respiratory capacity of microglial mitochondria (Figures 1G,H). The expression levels of OXPHOS and tricarboxylic acid (TCA) cycle genes were decreased in the brain of TRPV1^−/−^ mice compared with the WT group (Figures 1I,J). However, the ECAR analysis showed that glycolysis and glycolytic capacity were decreased in capsaicin treated BV2 cells compared to the control group (Figures 1K,L). Collectively, the results suggested that TRPV1 participate in autophagy and cellular energy metabolism in microglia.

### TRPV1 Activation Promotes Autophagic Flux and Phagocytosis in Microglia

**FIGURE 2:**
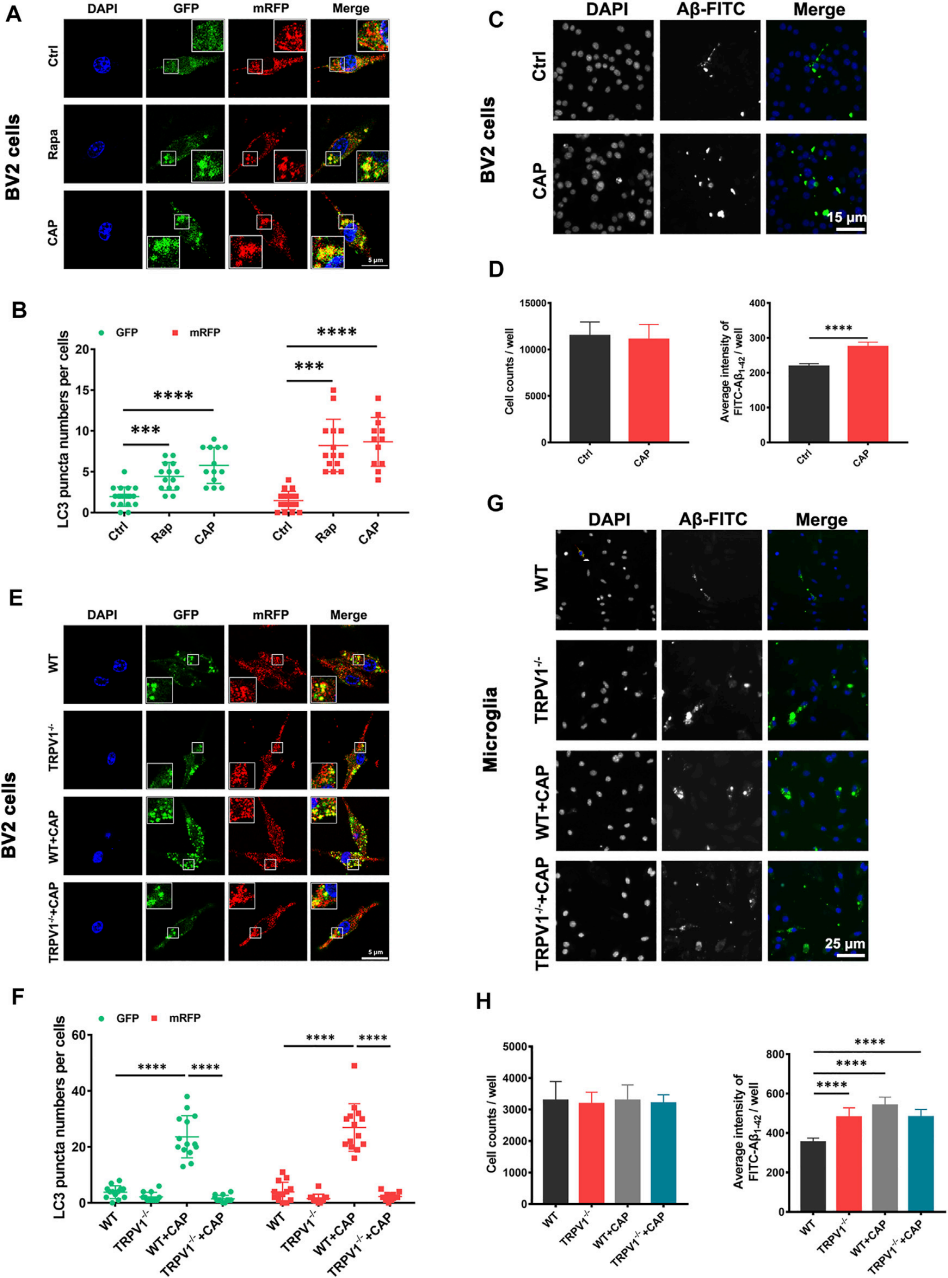
TRPV1 activation promotes autophagic flux and phagocytic capacity in microglia. **(A, B, E, F)** BV2 cells and primary microglia were transfected with mRFP-GFP-LC3 plasmids for 48 h. Representative confocal images of mRFP-GFP-LC3 location, the number of autophagosome dots (green), autolysosome and autolysosome dots (red) in BV2 cells **(A, B)** (*n* = 3, three independent experiments) and primary microglia **(E, F)** (*n* = 3, three independent experiments) after treated with 10 μM capsaicin or 2 μM rapamycin for 24 h. **(C, D, G, H)** Measurement of Aβ phagocytic capacity in BV2 cells or primary microglia. FITC-Aβ_1-42_ were added to the cells after incubated with 10 μM capsaicin for 24 h. Representative image **(C, G)** (*n* = 6, three independent experiments) and quantification of phagocytic capacity. **(D, H)** (*n* = 6, three independent experiments) using Cellomics KineticScan reader. Data are representative of three experiments. ^***^ *p* < 0.0001, ^****^ *p* < 0.0001, one-way ANOVA with the Tukey’s post-hoc test. Bars represent mean ± SEM. Scale bar for Panel **(A, E)**: 20 μm. Scale bar for Panel **(C)**: 15 μm. Scale bar for Panel **(G)**: 25 μm.

The mRFP-GFP-tagged LC3 reporter was introduced to further characterize the role of TRPV1 in microglial autophagy. The location of mRFP-GFP-LC3 was monitored in BV2 cells and primary microglia with 10 μM capsaicin or 2 μM autophagy activator rapamycin treatment (Figures 2A,B,E,F). GFP (green fluorescent protein) is sensitive to the acidic condition of the lysosome lumen, while mRFP (red fluorescent protein) signal is more stable (Luo et al., 2020). Capsaicin induced a significant upregulation of green and red puncta in both BV2 cells and primary microglia. Meanwhile, gene deletion of TRPV1 (TRPV1^−/−^) attenuated the autophagic flux in primary microglia with capsaicin treatment (Figures 2E,F). To investigate the implication of TRPV1 on microglial phagocytic capacity, the cells were incubated with 2 μg/ml FITC-Aβ_1-42_ for 4 h after pretreatment with 10 μM capsaicin for 24 h (Figures 2C,D,G,H). The phagocytic capacity was increased in both BV2 cells (Figures 2C,D) and primary microglia (Figures 2G,H) in the presence of capsaicin compared with control cells. This induction was completely abolished in TRPV1^−/−^-deficient microglia. These data indicate that capsaicin improved microglia energy metabolism, followed by the enhancement of immune function including autophagic flux and phagocytic capacity.

### TRPV1 Deficiency Curtails Energy Metabolism in Microglia via Autophagy Inhibition

**FIGURE 3:**
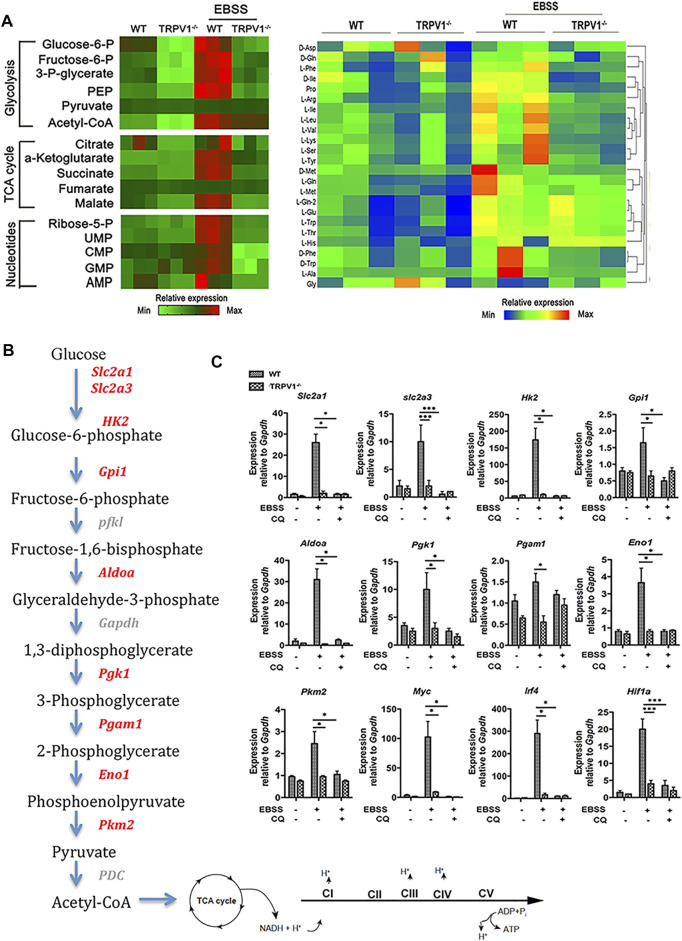
TRPV1 deficiency curtails energy metabolism in microglia via autophagy inhibition. **(A)** Analysis of polar metabolites including glycolytic, TCA-cycle intermediates, nucleotides, and amino acids by liquid chromatography–mass spectrometry in EBSS-stimulated autophagy of WT and TRPV1^−/−^ microglia. Heatmaps show relative metabolite concentrations per row in microglia repeated in triplicate. **(B, C)** TRPV1 regulates expression of glycolytic and mitochondrial enzymes. **(B)** Pathway map of glycolytic enzymes and the electron transport chain in mitochondria. **(C)** Expression of glycolytic enzymes and transcriptional regulators of glycolysis by real time-PCR in WT and TRPV1^−/−^ microglia before and 24 h after EBSS-stimulated autophagy in the presence or absence of 10 µM chloroquine (n = 3, three experiments). ^*^ *p* < 0.05, ^***^ *p* < 0.001; one-way ANOVA with the Tukey’s post-hoc test. Bars represent mean ± SEM. CQ, chloroquine.

To investigate how TRPV1 controls metabolism of microglia, polar-metabolite profiling of WT and TRPV1^−/−^ microglia was performed *via* liquid chromatography followed by mass spectrometry to capture the intermediates of glycolysis and the TCA cycle. In brief, microglia were incubated with EBSS to induce autophagy. As shown in Figure 3A, the levels of glycolytic metabolites were increased in EBSS-stimulated WT microglia as compared to the control cells, while TCA cycle intermediates were markedly increased. Moreover, there was a greater abundance of metabolites generated by the pentose phosphate pathway, which is crucial for the synthesis of nucleotides and amino acids in EBSS-stimulated microglia (Figure 3A). Stimulation of TRPV1^−/−^ cells resulted in a sharp decrease in the amounts of polar metabolites, such as glucose 6-phosphate (Figure 3A). Consequently, there were notable reductions in the amounts of nucleotides and amino acids in TRPV1^−/−^ cells (Figure 3A).

Stimulation of WT microglia caused pronounced upregulation of not only Slc2a1 and Slc2a3, but also several glycolytic enzymes, including hexokinase 2 (Hk2), aldolase A (Aldoa), phosphoglycerate kinase 1 (Pgk1), and enolase 1 (Eno1) (Figures 3B,C). This induction was completely abolished in TRPV1^−/−^-deficient microglia and microglia treated with autophagy inhibitor chloroquine (Figures 3B,C). The transcription of many of these enzymes is controlled by metabolic regulators including c-Myc, HIF-1a, and IRF4. We found that upon EBSS-stimulation induced autophagy, transcripts of Myc, Irf4, and Hif1a were strongly induced in WT cells but not in TRPV1^−/−^-deficient or chloroquine-treated microglia (Figures 3B,C).

### TRPV1 Activation Reversed Learning and Memory Impairment of 3×Tg AD Mice by Improving Autophagy

**FIGURE 4:**
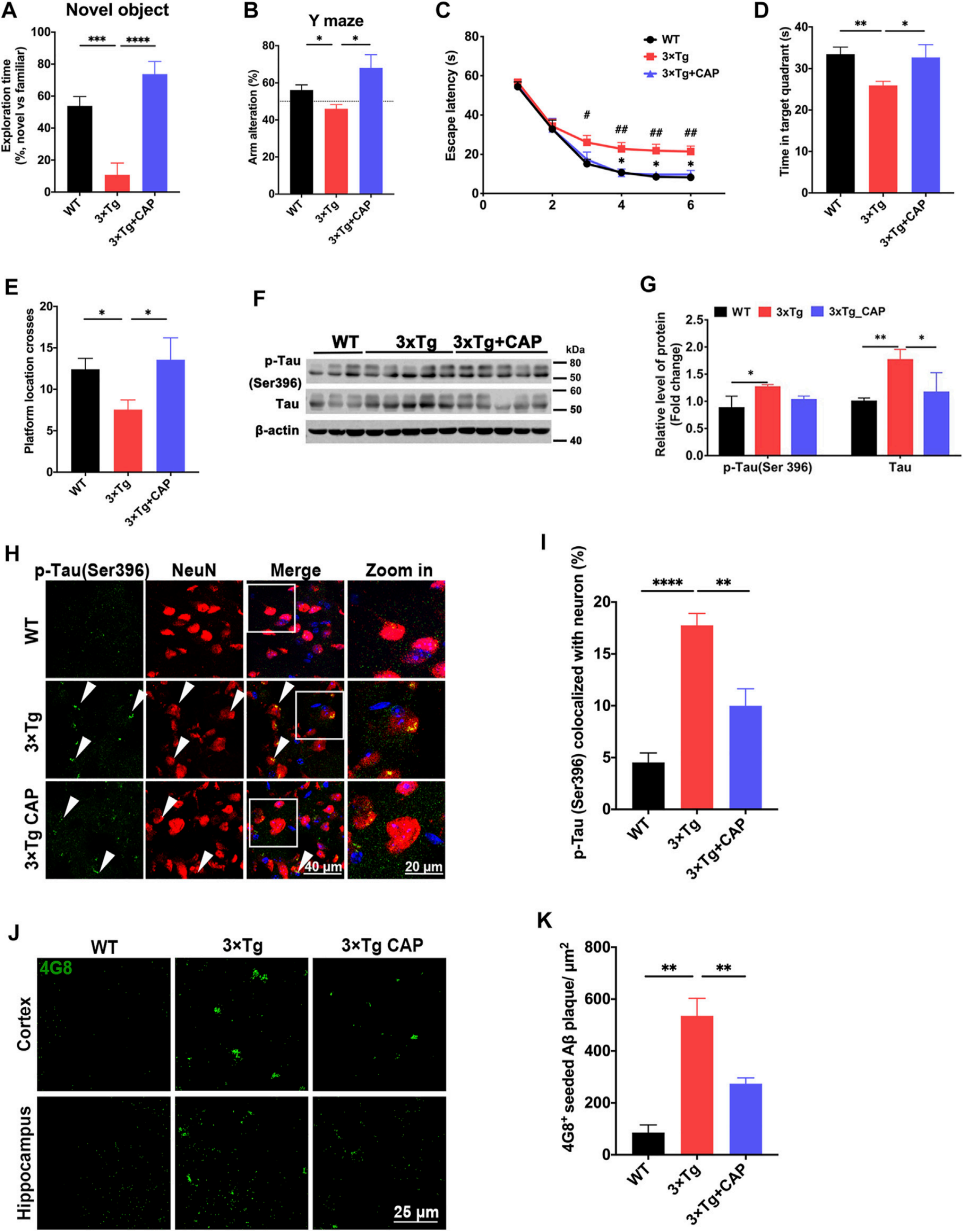
Capsaicin treatment reduced phosphorylated tau accumulation and β-amyloid plaque deposition in 3×Tg mice. **(A, B)** Histograms depicted exploration time to new object and new arm in novel object recognition test **(A)** and Y maze spontaneous alteration test **(B)** (n = 9 mice in WT group, *n* = 8 mice in 3xTg group, and *n* = 5 mice in 3xTg + capsaicin group). **(C–E)** Morris water maze behavioral assessment showed the escape latency **(C)**, time in target quadrant **(D)**, and platform crosses **(E)** (*n* = 6 mice in WT group, *n* = 8 mice in 3xTg group, and *n* = 5 mice in 3xTg + capsaicin group). **(F)** Representative immunoblot images and **(G)** quantifications of p-Tau (Ser396) and total tau (*n* = 3 mice in WT group, *n* = 5 mice in 3xTg group, and *n* = 5 mice in 3xTg + capsaicin group). **(H)** Representative immunofluorescence images showed the colocalization of p-tau (Ser396) and NeuN^+^ neuron. **(I)** Quantification of colocalization using Manders’ Correlation Index (*n* = 3 mice in each group; 5 fields from each mouse). **(J)** Representative immunofluorescence images showed 4G8^+^ β-amyloid plaque in the cortex (left) and hippocampus (right). **(K)** Quantification of 4G8^+^ Aβ plaque area (*n* = 3 mice in each group; 10 fields from each mouse). Data are shown as mean ± SEM. Statistical analysis used one-way ANOVA with Tukey’s multiple comparison test. ^*^ *p* < 0.05, ^**^ *p* < 0.01, ^***^ *p* < 0.001, ^****^ *p* < 0.0001 in **(A, B, D, E, G, I, K)**. ^#^ *p* < 0.05, ^##^ *p* < 0.01 compared to the WT group, and ^*^ *p* < 0.05 compared to the 3×Tg group in **C**. Scale bar: 50 μm **(H)**, 25 μm **(I)**. Scale bar for the high magnification insets: 20 μm.

Capsaicin treatment was shown to promote Aβ clearance in primary microglia *in vitro* (Figure 2), and then 7-month-old 3xTg mice were treated with capsaicin (1 mg/kg, intraperitoneally; a single injection/day) for 1 month. The therapeutic efficacy of the TRPV1 agonist capsaicin was examined in 8-month-old 3xTg mice. 3xTg mice showed significant impairment of learning and memory ability at 8-months-old compared to age-matched WT mice (Figures 4A–E), which was consistent with previous studies (Oddo et al., 2003; Belfiore et al., 2019). Capsaicin treatment significantly reversed impaired recognition to novel object or novel area in novel object recognition assay (Figure 4A) and Y maze spontaneous alteration test (Figure 4B). A probe trial was conducted in Morris water maze test after a 6-days’ training session to evaluate memory ability in regard to a spatial reference (Figure 4C). Memory was strongly impaired in 3xTg mice as compared to the WT group (Time in the target quadrant; Figure 4D). The number of platform crosses was increased in the 3xTg + capsaicin group as compared to the 3xTg group (Figure 4E). These results suggested that capsaicin treatment ameliorated 3xTg mice neurodegeneration in learning and memory.

AD pathology including β-amyloid accumulation and hyperphosphorylated tau was further observed to evaluate the therapy effect of capsaicin in 3xTg mice. Immunoblotting showed upregulation of total tau and phosphorylated tau in 3xTg mice compared to WT mice. Capsaicin significantly decreased the expression of both phosphorylated tau and total tau in 3xTg + capsaicin group mice compared to 3xTg group mice (Figures 4F,G). Immunofluorescence of brain sections also showed a significant decrease of both phosphorylated tau (Ser396) (Figures 4H,I) and 4G8^+^ amyloid plaque accumulation (Figures 4J,K) in 3xTg + capsaicin group mice (Figures 4H–K). Those results suggested that capsaicin treatment on 3×Tg mice reversed their neurodegeneration process by decreasing the accumulation of β-amyloid and phosphorylated tau.

### TRPV1 Activation Rescued Impaired Energetic Metabolism and Autophagy Function in the Brain of AD Mice Model

**FIGURE 5:**
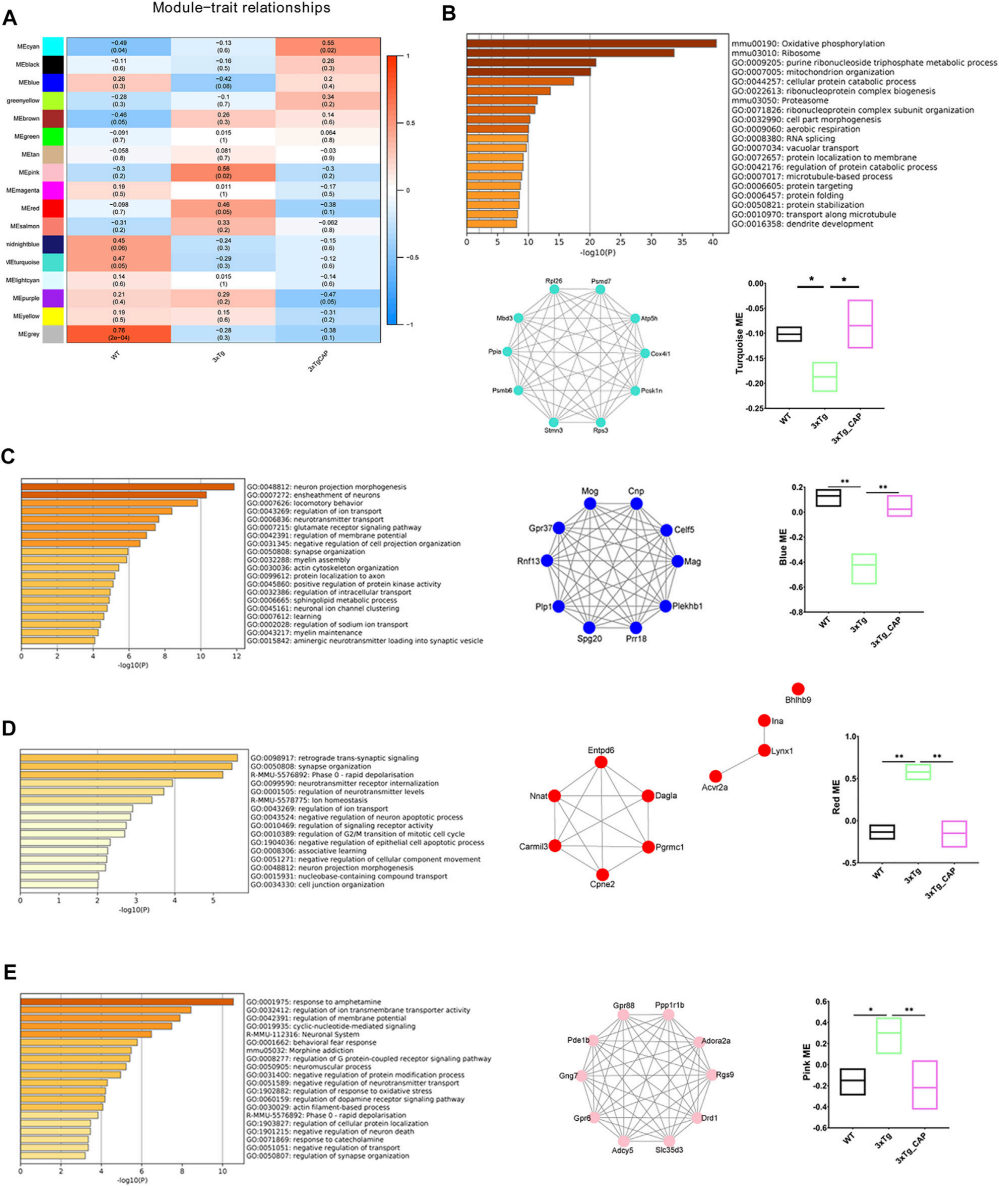
WGCNA of 3xTg mice brains with capsaicin treatment. **(A)** Modules associated with the groups of WT, 3xTg, and 3xTg + capsaicin group mice (*n* = 4 in 3xTg and 3xTg + capsaicin mice, *n* = 3 in WT mice). Numbers on the heatmap show the correlation coefficient. Modules with positive values (orange) indicate upregulation in the corresponding group compared to others while negative values (blue) indicate downregulation. **(B–E)** Bar graphs show the top 20 pathways of GO and KEGG enrichment analysis of the turquoise **(B)**, blue **(C)**, red **(D)**, and pink **(E)** module (*n* = 4 in 3xTg and 3xTg + capsaicin mice, *n* = 3 in WT mice). Network plot graphs show the top 10 hub genes with highest intramodular connectivity. Box plot graphs show trajectory of the MEs among the three groups involved. Statistical analysis used one-way ANOVA with Tukey’s multiple comparison test. ^*^ *p* < 0.05, ^**^ *p* < 0.01.

To identify the effects of activating TRPV1 on AD brain, we injected capsaicin (1 mg/kg, intraperitoneally; a single injection/day) for 1 month on 7-month-old 3xTg mice. RNA sequencing was performed to determine the phenotype of WT, 3xTg, or 3xTg + capsaicin brain transcriptionally. WGCNA showed that four major modules identified dysregulation in 3xTg group mice. Two upregulated modules (turquoise and blue) and two downregulated modules (red and pink) were detected in 3xTg + capsaicin group mice compared to 3xTg group mice (Figure 5A). Enrichment analyses revealed the improvement of oxidative phosphorylation, mitochondrion organization, neuron and synapse function of 3xTg mice treated with capsaicin, as well as locomotory behavior (Figures 5B,C). Capsaicin treatment also leads to decrease the levels of cell apoptotic process and response to oxidative stress (Figures 5D,E). Those results showed ameliorated effects of capsaicin treatment by both improving cellular OXPHOS and mitochondrion function, leading to improvement of neuronal and synapse functions transcriptionally. OXPHOS plays critical roles in brain energy metabolism, and OXPHOS dysfunction may lead to multiple kinds of neurodegeneration including AD (Koopman et al., 2013; Baik et al., 2019).

### Genome-Wide RNA Sequencing of 3xTg Mice Reveals Treatment with Capsaicin Alleviates Pathological Hallmarks of AD in 3xTg Mice

**FIGURE 6:**
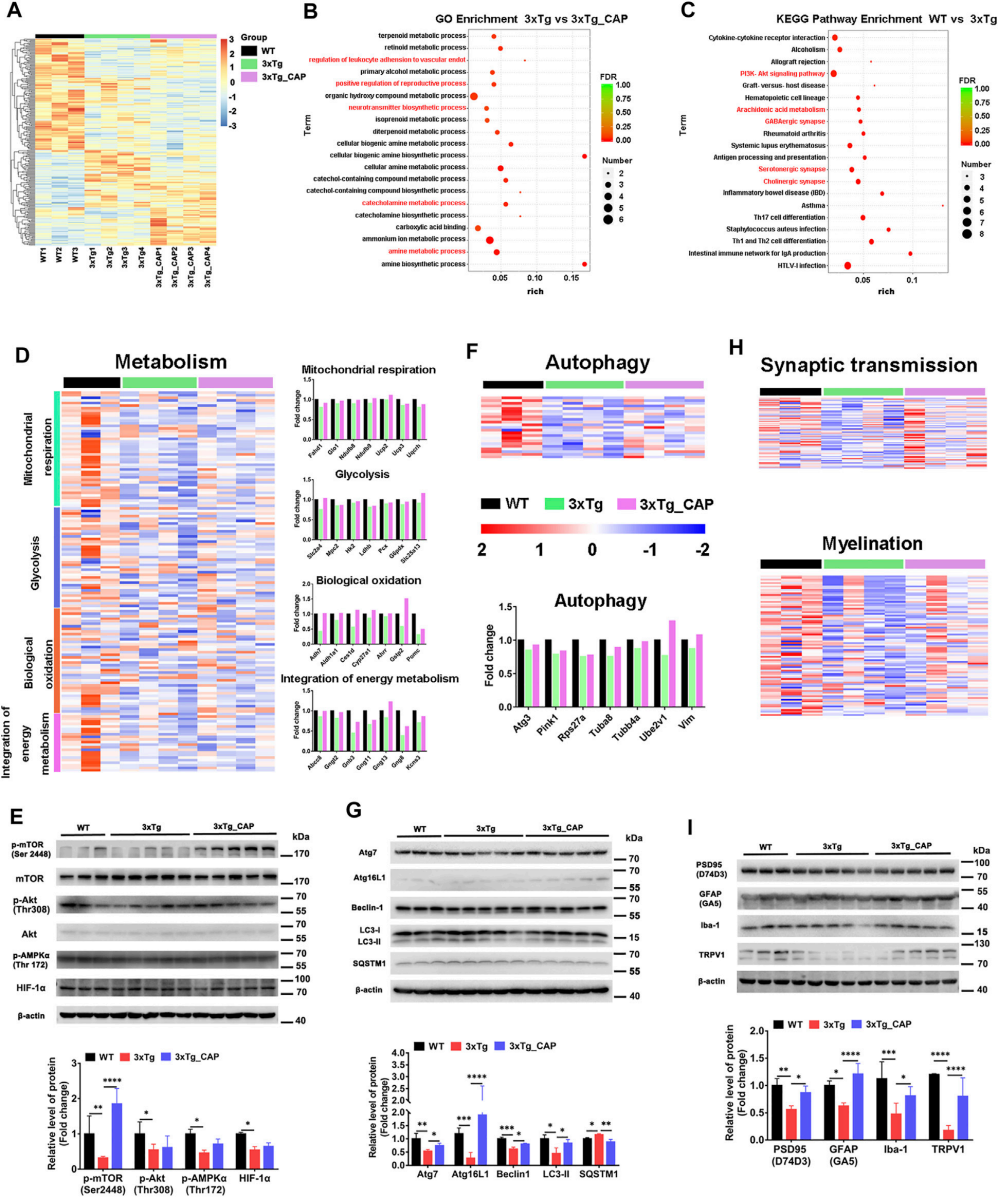
TRPV1 activation rescued impaired energetic metabolism and autophagy function in the brain of 3xTg mice. **(A)** A two-way cluster analysis of differential gene in WT, 3xTg, and 3xTg + capsaicin group mice (*n* = 4 in 3xTg and 3xTg + capsaicin mice, *n* = 3 in WT mice). **(B, C)** Differentially expressed genes analyzed by GO enrichment in brains of WT, 3xTg, and 3xTg + capsaicin mice (*n* = 4 in 3xTg and 3xTg + capsaicin mice, *n* = 3 in WT mice). **(D)** Heatmap depicts the metabolic profiles and bar graph shows the fold change of genes within the group (*n* = 4 in 3xTg and 3xTg + capsaicin mice, n = 3 in WT mice). **(E)** Western blot analysis of the protein level of AKT/mTOR/HIF-1α pathway (*n* = 5 in 3xTg and 3xTg + capsaicin mice, *n* = 3 in WT mice). **(F)** Heatmap represents the 23 differentially expressed autophagy-related genes and histogram displays the fold change of genes (*n* = 4 in 3xTg and 3xTg + capsaicin mice, n = 3 in WT mice). **(G)** Western blot analysis of the expression of autophagy-associated proteins (*n* = 5 in 3xTg and 3xTg + capsaicin mice, *n* = 3 in WT mice). **(H)** Heatmap of the expression levels of synaptic transmission and myelination genes (*n* = 4 in 3xTg and 3xTg + capsaicin mice, *n* = 3 in WT mice). **(I)** Western blot analysis of the expression of PSD95, GFAP, Iba-1, and TRPV1 (*n* = 5 in 3xTg and 3xTg + capsaicin mice, *n* = 3 in WT mice). Data are presented as mean ± SEM, ^*^ *p* < 0.05, ^**^ *p* < 0.01, ^***^ *p* < 0.001, ^****^ *p* < 0.0001, one-way ANOVA with Tukey’s multiple comparison test.

Hierarchical clustering of capsaicin-treated 3xTg AD mice brains revealed differences in brain transcriptomes that corresponded to TRPV1 status (Figure 6A). The transcriptomes included 20 differentially upregulated and 21 differentially downregulated genes in expression in 3xTg mice compared with 3xTg + capsaicin mice. The 20 upregulated genes with the greatest differences in expression were listed in Table 2. The 19 genes with the greatest degree of downregulation were listed in Table 3. The GO Consortium classification (http://www.godatabase.org/cgi-bin/amigo/go.cgi) biological process- and class-associated genes in 3xTg and 3xTg + capsaicin mice are shown in Figure 6B. The most prominent were genes that function in metabolic process, biosynthetic process, corresponding to genes related to reactions in response to the decreased of nutrients and neurotransmitter in brain. The 20 most enriched KEGG pathways in the WT vs. 3xTg mice are shown in Figure 6C, which related to PI3K-AKT signaling pathway, GABAergic synapse, serotonergic synapse, cholinergic synapse, and antigen processing and presentation.

**TABLE 2:** Top 20 upregulated genes in capsaicin-treated 3xTg AD mice compared with 3xTg AD mice

ID	Name	3xTg	3xTg- CAP	Fold change
ENSMUSG00000032484	Ngp	0.39	3.21	8.34
ENSMUSG00000041566	Tssk1	0.80	4.71	5.87
ENSMUSG00000025496	Drd4	1.94	7.45	3.83
ENSMUSG00000028003	Lrat	2.33	8.05	3.46
ENSMUSG00000026774	Potegl	1.94	6.43	3.31
ENSMUSG00000041062	Mslnl	2.44	7.84	3.22
ENSMUSG00000059430	Actg2	2.15	6.70	3.12
ENSMUSG00000078139	AK157302	3.32	9.94	3.00
ENSMUSG00000000889	Dbh	2.52	7.49	2.97
ENSMUSG00000006574	Slc4a1	3.30	9.63	2.92
ENSMUSG00000032845	Alpk2	3.14	8.47	2.70
ENSMUSG00000074489	Bglap3	5.21	13.21	2.54
ENSMUSG00000021214	Akr1c18	10.74	25.46	2.37
ENSMUSG00000023903	Mmp25	4.93	11.67	2.37
ENSMUSG00000027360	Hdc	35.09	77.35	2.20
ENSMUSG00000029134	Plb1	12.15	26.22	2.16
ENSMUSG00000048489	Depp1	21.94	46.89	2.14
ENSMUSG00000040706	Agmat	11.54	24.56	2.13
ENSMUSG00000037405	Icam1	30.33	61.40	2.02
ENSMUSG00000025355	Mmp19	8.09	16.29	2.01

Shown are the top 20 genes in order of fold change with normalized linear expression in 3x Tg AD, mice.

**TABLE 3:** Top 19 downregulated genes in capsaicin-treated 3xTg AD mice compared with 3xTg AD mice

ID	Name	3xTg	3xTg- CAP	Fold change
ENSMUSG00000021416	Eci3	2.75	0.20	13.71
ENSMUSG00000043230	Fam124b	5.77	0.60	9.54
ENSMUSG00000108900	Ccdc194	4.96	0.81	6.14
ENSMUSG00000101578	Vmn1r206	9.10	1.61	5.65
ENSMUSG00000042377	Fam83g	4.47	1.03	4.35
ENSMUSG00000044201	Cdc25c	8.36	2.01	4.16
ENSMUSG00000004341	Gpx6	32.85	8.52	3.86
ENSMUSG00000013936	Myl2	5.28	1.40	3.76
ENSMUSG00000038651	Sycp2l	10.57	3.26	3.24
ENSMUSG00000009070	Rsph14	8.30	2.86	2.90
ENSMUSG00000024124	Prss30	10.30	3.67	2.81
ENSMUSG00000051663	Pcdhb1	13.03	5.06	2.57
ENSMUSG00000025271	Pfkfb1	18.03	7.28	2.48
ENSMUSG00000005131	4930550C14Rik	10.08	4.25	2.37
ENSMUSG00000054293	P2ry10b	11.24	4.91	2.29
ENSMUSG00000074651	Mcidas	12.26	5.70	2.15
ENSMUSG00000029847	Slc23a4	14.08	6.70	2.10
ENSMUSG00000074715	Ccl28	64.48	31.02	2.08
ENSMUSG00000041673	Lrrc18	14.79	7.35	2.01

Shown are the top 19 genes in order of fold change with normalized linear expression in 3x Tg AD, mice.

**FIGURE 7:**
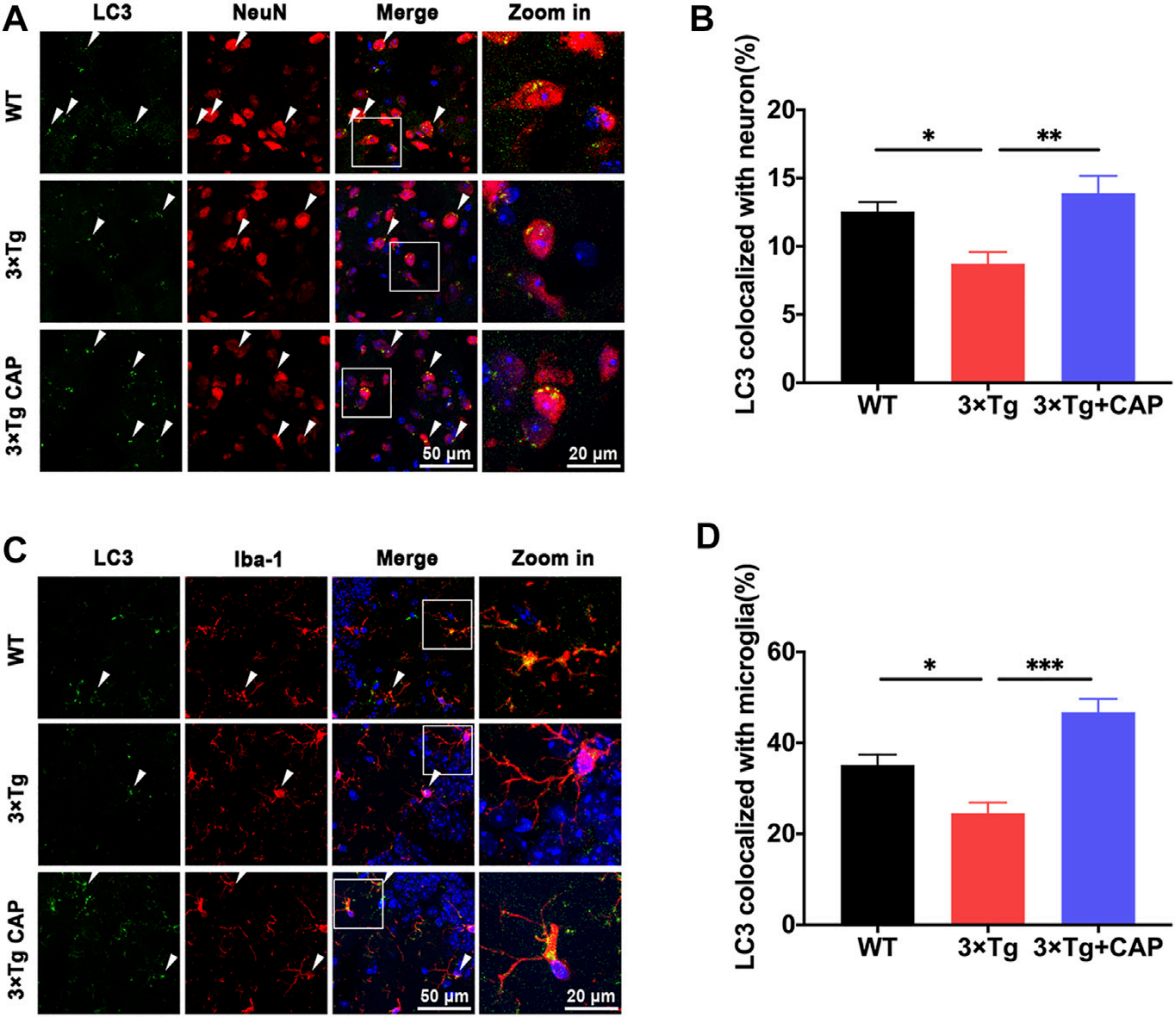
Histograms depicted mRNA levels of different crucial receptor markers Slc17a6, Slc17a7, Chat, and Gad1 of WT, 3xTg, and 3xTg+capsaicin mice (*n* = 4 mice in 3xTg and 3xTg + capsaicin mice, *n* = 3 in WT mice). Data are presented as mean ± SEM, ^*^ *p* < 0.05, ^**^ *p* < 0.01, one-way ANOVA with Tukey’s multiple comparison test.

Hierarchical clustering generated the heat maps of genes that regulate energy metabolic transcripts (Figure 6D) and autophagy (Figure 6F) which were found to have significantly increased expression in 3xTg + capsaicin mice compared to 3xTg mice. Immunoblotting of total lysates from the mice brains showed the upregulation of energy metabolic AKT-mTOR-HIF-1α signaling pathway (Figure 6E) and autophagic markers (Figure 6G) which are consistent with the heatmap in each group. Finally, we analyzed the genes associated with synaptic transmission and myelination according to the prompts of enriched biological processing (Figure 6H). The heatmap depicted downregulated genes expression in 3xTg mice compared to WT mice, and capsaicin treatment on 3xTg mice upregulated synaptic transmission associated genes expression compared to 3xTg mice. To investigate the changes on different kinds of synaptic receptors, mRNA levels of cholinergic, glutamatergic, and GABAergic receptors were detected (Konstantinides et al., 2018). mRNA levels of glutamatergic receptor markers Slc17a6 (vesicular glutamate transporter 2, Vglut2) and Slc17a7 (Vglut1) were also downregulated in 3xTg mice compared to WT ones, and 3xTg + capsaicin mice showed more gene expression than 3xTg mice (Figure 7). Previous study revealed decreased Vglut in AD model mice (Yeung et al., 2020). In addition, there was no difference of cholinergic receptor marker choline acetyltransferase (Chat) or GABAergic receptor marker glutamic acid decarboxylase 1 (Gad1) among those three groups of mice (Figure 7).

Immunoblotting showed the protein levels of postsynaptic density protein 95 (PSD95) and TRPV1 were upregulated in 3xTg + capsaicin mice compared to 3xTg mice. Microglia-specific marker ionized calcium binding adaptor molecule 1 (Iba1), an evolutionarily conserved cytoplasmic calcium binding protein, is upregulated in activated microglia (Imai and Kohsaka, 2002). Upregulated expression of glial fibrillary acidic protein (GFAP) is considered as a feature of activated astrocytes (Dubový et al., 2018). The reactivation of microglia and astrocytes were rescued from the immune tolerant status in 3xTg mice (Figure 6I). These data demonstrate that brain of 3xTg AD transgenic mice were immune tolerant and metabolically defected *in vivo* compared with those of WT mice and suggested that treatment with capsaicin rescued pathological hallmarks of AD in 3xTg mice by promoting glial activation, metabolism, and autophagy.

**FIGURE 8:**
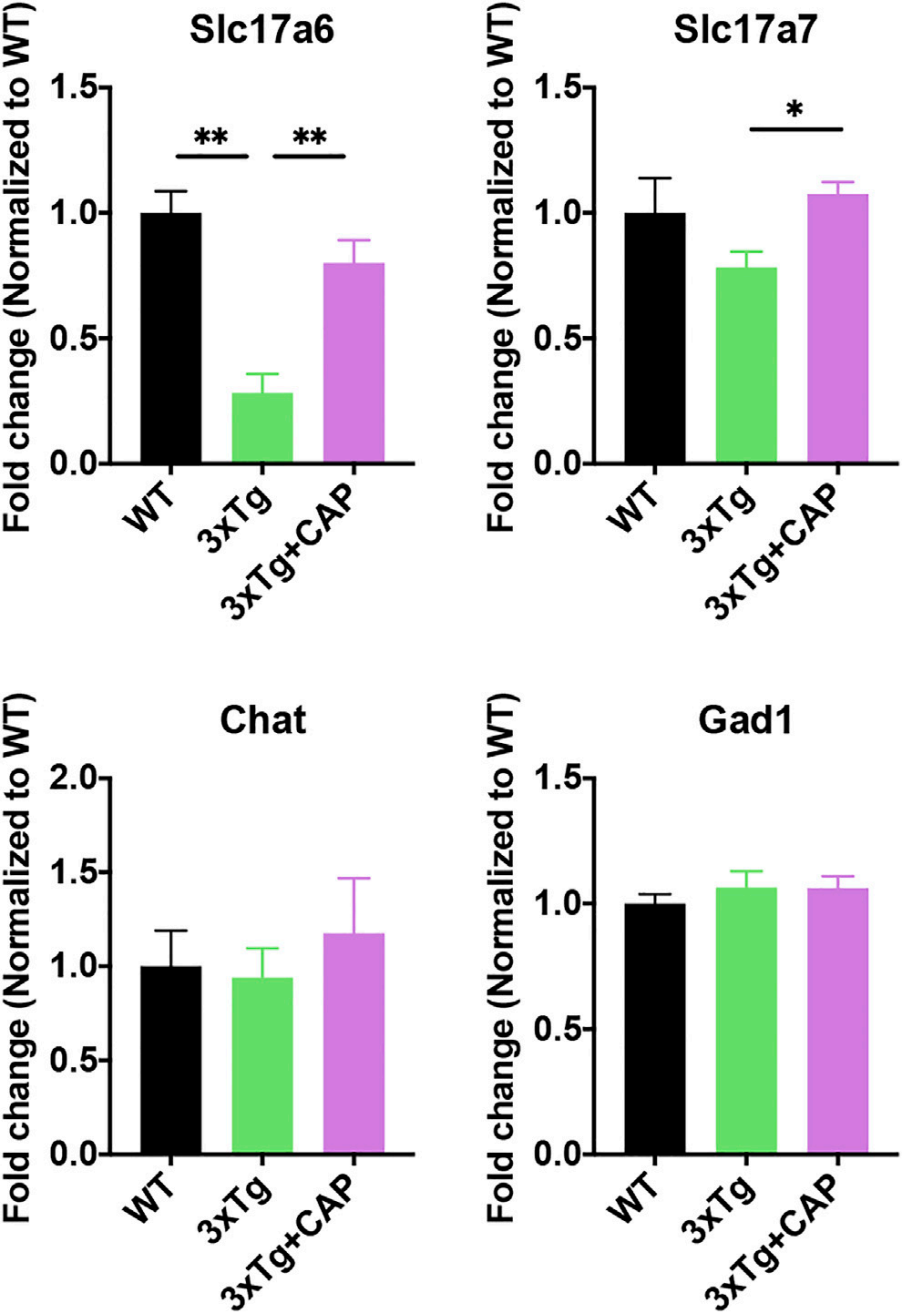
TRPV1 activation rescued microglial neuroimmune suppression in 3xTg mice. **(A, B)** Histograms depicted quantitative analysis of average branch length **(A)** and number of end-point voxels **(B)** of Iba-1^+^ microglia. (C) Heatmap represented 320 differentially expressed microglial neuroimmune genes. **(D)** Z-score of genes from neuroimmune suppression cluster for all groups. **(E)** Histogram depicted top differentially expressed neuroimmune suppression cluster genes from the heatmap (criteria: *p* value of 3xTg and 3xTg + capsaicin low to high). *n* = 3 mice in each group. Data are presented as mean ± SEM, ^*^ *p* < 0.05, ^**^ *p* < 0.001, ^****^ *p* < 0.0001. Statistical tests: one-way ANOVA with Tukey’s multiple comparison test.

To further investigate microglial activation of 3xTg mice with capsaicin treatment, morphological analysis of microglia was performed. Microglia of 3xTg mice showed more end-point voxels and longer branch length than WT mice, which indicated a less activated state of microglia (Figures 8A,B) (Bolmont et al., 2008; Baron et al., 2014; Plescher et al., 2018). The number of end-point voxels and microglial branch length in capsaicin-treated 3xTg mice decreased compared to 3xTg mice (Figures 8A,B), which indicated microglial activation in capsaicin-treated 3xTg mice. Moreover, transcriptome profiles of genes related to microglial immune functions were further analyzed. A total of 320 genes related to microglial neuroimmune suppression were involved (Rexach et al., 2020). Results depicted upregulation of neuroimmune suppression genes in 3xTg mice compared to WT mice, which indicated immune suppression of microglia in 3xTg mice (Figures 8C–E). Neuroimmune suppression cluster genes decreased in capsaicin-treated 3xTg mice brain compared to 3xTg mice (Figures 8C–E).

**FIGURE 9:**
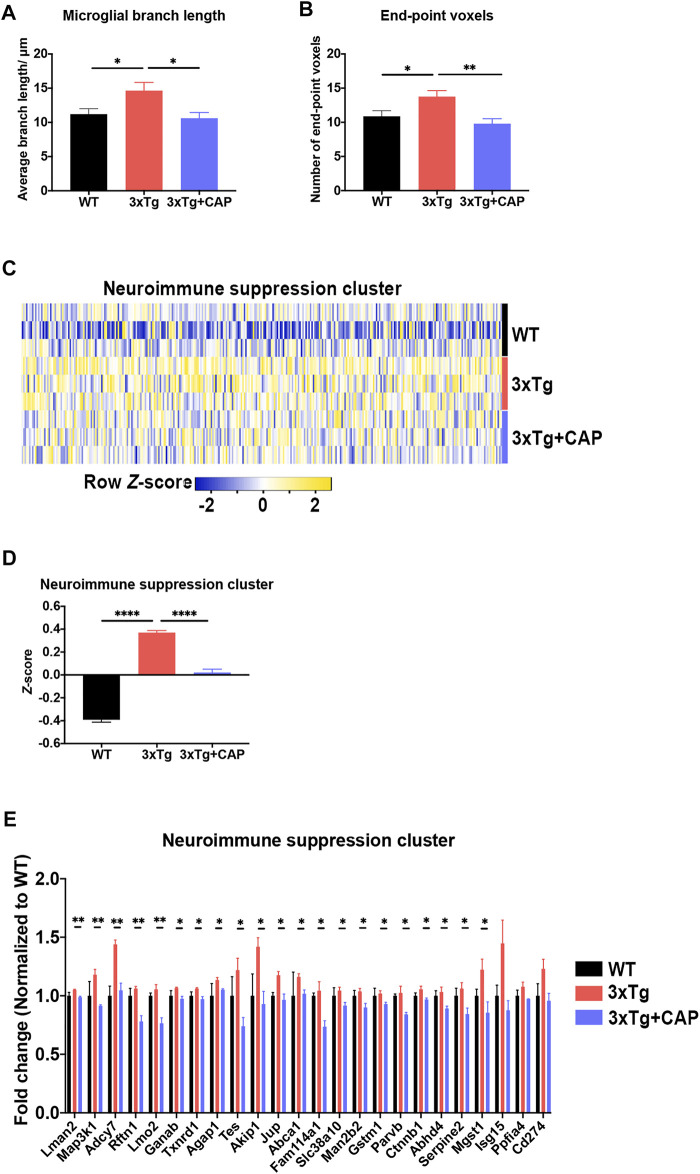
Capsaicin reversed autophagy decrease in both microglia and neurons on 3×Tg mice. **(A, C)** Representative images showing the colocalization of LC3B/NeuN **(A)** or LC3B/Iba-1 **(C)**. **(B, D)** Quantification of colocalization of LC3B/NeuN **(B)** or LC3B/Iba-1 **(D)** using Manders’ Correlation Index. Each point represented one field from three mice. *n* = 3 mice in each group; 5 fields from each mouse. Data are shown as mean ± SEM. Statistical analysis used one-way ANOVA with Tukey’s multiple comparison test. ^*^ *p* < 0.05, ^**^ *p* < 0.01, ^***^ *p* < 0.001. Scale bar: 50 μm. Scale bar for the high magnification insets: 20 μm.

The assessment of autophagy protein levels was also performed by immunofluorescence. As shown in Figure 9, the colocalization of LC3B/Iba-1 (Figures 9A,B) or LC3B/NeuN (Figures 9C,D) were significantly decreased in 8-month-old 3×Tg mice compared to WT mice. Capsaicin treatment significantly increased the colocalization of LC3B/Iba-1 or LC3B/NeuN, which suggested improving autophagy levels of neuron and microglia in 3xTg + capsaicin group mice (Figures 9A–D).

## Discussion

The results of the present study provide both *in vitro* and *in vivo* evidence that TRPV1-mediated autophagy reduced amyloid and phosphorylated tau pathology and attenuated cognitive decline in a murine model. First, the TRPV1 agonist capsaicin induced autophagy lysosome biogenesis in microglia, which was abolished via gene deletion. TRPV1 regulated the microglia metabolic process by controlling the expression of genes required for aerobic glycolysis and mitochondrial respiration. TRPV1 agonist capsaicin decreased amyloid and phosphorylated tau pathology and reversed memory deficits by promoting microglia activation, metabolism, and autophagy in 3xTg mice.

TRPV1 receptor was cloned in 1997 from the rat dorsal root ganglia, which was described as a specific target of capsaicin and resiniferatoxin (Xu et al., 1997). TRPV1 channel can be activated not only by endogenous compounds such as endocannabinoids and arachidonic acid metabolites, but also by exogenous agonist capsaicin (Mori et al., 2011). TRPV1 activation alleviates cognitive and synaptic plasticity impairments through inhibiting AMPAR endocytosis in APP23/PS45 mouse model of AD (Du et al., 2020).

TRPV1 is involved in the pathophysiology of conditions including chronic inflammatory pain, asthma, cystitis, diabetes-associated peripheral neuropathy, and hearing loss. Pharmacological modulation of TRPV1 channel has potential for treatment of these conditions. In our study, 3xTg AD mice model was used to find that activation of Ca^2+^-permeable TRPV1 attenuated memory deficits and the accumulation of Aβ and phosphorylated tau. Capsaicin, the TRPV1 agonist, might have therapeutic potential for AD treatment.

TRPV1 reportedly protects against foam cell formation in oxidized low-density lipoprotein-treated vascular smooth muscle cells through the induction of autophagy (Li et al., 2014). Autophagy sustains cell survival under nutrient deprivation conditions and the removal of aggregated proteins and damaged cellular organelles to maintain homeostasis. In mammalian cells, three main types of autophagy exist, including macroautophagy, microautophagy, and chaperone-mediated autophagy, which is a complex process involving many ATGs (Mizushima and Komatsu, 2011; Settembre et al., 2013). The abundance of autophagy-related proteins, such as Beclin1, ATG5, and ATG7, declines with age and is exacerbated during AD (Pickford et al., 2008; Lipinski et al., 2010; Rubinsztein et al., 2011; Lucin et al., 2013). Autophagy has been explored in the context of alterations to the function and homeostasis of neurons, which leads to neuronal dysfunction. While autophagy of the neuron has been implicated as a protective role in the early stages of AD (Maday, 2016), autophagy-related proteins are less well-characterized in microglia and brain immune activation.

Previous studies showed that 10 μM capsaicin or 2 μM rapamycin treatment for 24 h could alleviate microglial activation and Aβ generation in SH-SY5Y-APP695 cells (Bhatia et al., 2017; Luo et al., 2020; Shibata et al., 2020; Wang et al., 2020). We showed that pharmacological activation of TRPV1 with capsaicin attenuated the accumulation of phosphorylated tau and total tau (Figures 4F–I). Capsaicin treatment in 3xTg mice showed a significant upregulation of energy metabolic mTOR/Akt/AMPK/HIF-1a pathway (Figure 6E) and autophagic markers Atg7, Atg16L1, Beclin-1, and LC3 (Figure 6G) as compared to 3xTg mice. Capsaicin treatment attenuated phosphorylated tau and Aβ accumulation in 3xTg mice by promoting glial activation, metabolism, and autophagy (Figure 6I, Figure 9C).

Metabolic transcripts were measured including mitochondrial respiration, glycolysis, biological oxidation, and integration of energy metabolism (Figure 6D). The anionic carrier protein uncoupling protein 2 is mainly involved in the uncoupling of the mitochondrial proton transport chain. Hexokinase 2 is an enzyme that catalyzes the phosphorylation of hexose, which is the rate-limiting enzyme in the glycolysis pathway (Figure 6D). Autophagy-related gene3 encodes a ubiquitin-like conjugating enzyme, which is a component of the ubiquitination-like system involved in autophagy, degradation, turnover, and recycling of cytoplasmic components (Figure 6F). PTEN-induced putative kinase 1 (PINK1) is a protein kinase, which participated in mitochondrial autophagy, and is mainly located in the inner membrane of mitochondria to protect mitochondria.

Previous studies revealed that capsaicin showed both dose-dependent and dose-independent effects in hippocampal neuronal network performance in AD model and *in vitro* (Balleza-Tapia et al., 2018; Balleza-Tapia et al., 2020). Shibata et al. has reported that capsaicin-induced autophagy was a dose-dependent process in neuronal cells, and our results were in line with previous findings (Shibata et al., 2020). Moreover, mechanisms of capsaicin also included TRPV1-independent pathway (Balleza-Tapia et al., 2018), and effects of capsaicin on AD need more research in the future.

In conclusion, the results of the present study demonstrated that activation of TRPV1 improved spatial learning and memory impairments in 3xTg mice via TRPV1-mediated autophagy and lysosomal pathways and activation of microglia, which together induced Aβ clearance. Based on these data, activation of TRPV1-mediated autophagy might be a promising therapeutic target against AD.

## Data Availability

The datasets presented in this study can be found in online repositories. The names of the repository/repositories and accession number(s) can be found here: https://ngdc.cncb.ac.cn/gsa/, CRA004946.

## Abbreviations

AD, Alzheimer’s disease; DAPI, 4′,6-diamidino-2-phenylindole, dihydrochloride; EBSS, Earle’s balanced salts solution; ECAR, extracellular acidification rate; FITC, fluorescein isothiocyanate; Aβ, amyloid-β; GO, gene ontology; KEGG, Kyoto Encyclopedia of Genes and Genomes; OXPHOS, oxidative phosphorylation; 2-DG, 2-deoxy-glucose; OCR, oxygen consumption rate; MEs, module eigengenes; PBS, phosphate-buffered saline; TCA, tricarboxylic acid; TRPV1, transient receptor potential vanilloid type 1; WGCNA, weighed gene co-expression network analyses.
